# Personalized analysis of minimal residual cancer cells in peritoneal lavage fluid predicts peritoneal dissemination of gastric cancer

**DOI:** 10.1186/s13045-021-01175-2

**Published:** 2021-10-12

**Authors:** Dongbing Zhao, Pinli Yue, Tongbo Wang, Pei Wang, Qianqian Song, Jingjing Wang, Yuchen Jiao

**Affiliations:** 1 Department of Pancreatic and Gastric Surgical Oncology, National Cancer Center/National Clinical Research Center for Cancer/Cancer Hospital, Chinese Academy of Medical Sciences and Peking Union Medical College, Beijing, 100021 China; 2 State Key Laboratory of Molecular Oncology, National Cancer Center/National Clinical Research Center for Cancer/Cancer Hospital, Chinese Academy of Medical Sciences and Peking Union Medical College, Panjiayuan nanli 17, Chaoyang District, Beijing, 100021 China; 3 Jinchenjunchuang Clinical Laboratory, Hangzhou, Zhejiang China

**Keywords:** Gastric cancer, Peritoneal lavage fluid, Peritoneal dissemination, Personalized mutation assay, Minimal residual disease

## Abstract

Peritoneal dissemination (PD) is a major type of gastric cancer (GC) recurrence and leads to rapid death. Current approaches cannot precisely determine which patients are at high risk of PD to provide early intervention. In this study, we developed a technology to detect minimal residual cancer cells in peritoneal lavage fluid (PLF) samples with a personalized assay profiling tumor-specific mutations. In a prospective cohort of 104 GC patients, the technology detected all the cases that developed PD with 100% sensitivity and 85% specificity. The minimal residual cancer cells in PLF were associated with a significantly increased risk of PD (HR = 145.13; 95% CI 20.20–18,435.79; *p* < 0.001), which was the strongest independent predictor over pathologic diagnosis and cytological diagnosis. In pathologically high-risk (pT4) patients, the PLF mutation profiling model exhibited a greater specificity of 91% and a positive predictive value of 88% while retaining a sensitivity of 100%. This approach may help in the postsurgical management of GC patients by detecting PD far before metastatic lesions grow to a significant size detectable by conventional methods such as MRI and CT scanning.

**Supplementary Information:**

The online version contains supplementary material available at 10.1186/s13045-021-01175-2.

## To the Editor,

Peritoneal dissemination (PD) is a major type of gastric cancer (GC) recurrence and a strong indicator of poor prognosis [1, 2]. Although multiple therapeutic solutions such as hyperthermic intraperitoneal chemotherapy (HIPEC) have been developed to prevent PD [3–5], current diagnostic approaches cannot precisely determine which patients will develop PD. When detectable by CT/MRI or causing symptoms, PD lesions are often of significant size, with no effective treatments available. Minimal residual disease (MRD) detection based on tumor-specific mutations in plasma cell-free DNA (cfDNA) has shown promising performance in prognostic prediction and disease monitoring in several tumor types, including breast, colorectal and lung cancers [6–10].

**Fig. 1:**
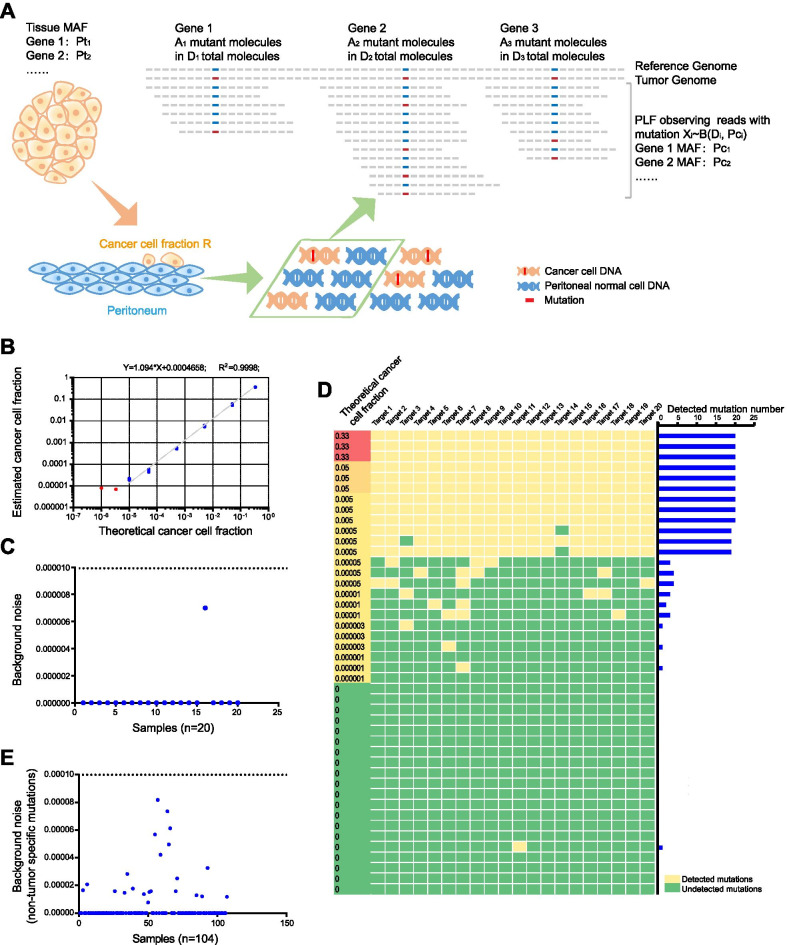
Cancer cell fraction model and background noise. **a** Cancer cell fraction model. A model for estimating the cancer cell fraction based on allele frequency and sequencing depth of somatic mutations in tumor tissue and paired PLF samples. MAF: mutant allele frequency; *Pt*_*i*_ : MAF in solid tumor tissue; *Pc*_*i*_ : MAF in corresponding peritoneal lavage fluid (PLF); *D*_*i*_ : sequencing depth in PLF; *A*_*i*_ : mutation read number in PLF; *X*_*i*_ : observed reads with a mutation in PLF; and R: overall cancer cell fraction. **b** The linear correlation between the theoretical and estimated cancer cell fractions. Each dilution was repeated three times. The blue dots highlight the fractions above the limit of detection (PLC/PRF/5 cell fraction = 0.001%, 0.005%, 0.05%, 0.5%, 5% and 33%). The red dots highlight the fractions under the limit of detection (PLC/PRF/5 cell fraction = 0.0001%, 0.0003%). **c** Background noise observed in the cancer cell fraction model at 0% PLC/PRF/5 cell input among the 20 independent replicates. **d** The distribution of mutations detected in the cancer cell fraction model. We profiled 20 SNPs to calculate the estimated dilution ratio with the model. Left panel: Heatmap illustrating detected (yellow) and undetected mutations (green) for each dilution. Right panel: the number of detected mutations. **e** Biological noise of the 104 PLF samples from patients. The cancer cell fraction for each sample was calculated based on nontumor-specific mutations

Here, we developed customized mutation profiling technology to detect minimal residual cancer cells from peritoneal lavage fluid (PLF). For each case, 20 tumor-specific mutations were selected from exome sequencing of the tumor tissue, and a personalized assay based on Mutation Capsule, a mutation profiling technology, was developed to detect the mutations. The assay was applied to the genomic DNA from cell pellets in the matched PLF samples, which were collected after abdominal exploration and before any manipulation of the stomach in the surgery. A model was developed to determine the fraction of cancer cells among normal cells in PLF based on the number and fraction of the mutations detected (Fig. 1a). The materials and methods are shown in detail in Additional file 1.

To validate the accuracy of the assay, we made a standard reference with serial dilutions of PLC/PRF/5 cells into A549 cells (Additional file 2: Table S1). We selected 20 SNPs unique to PLC/PRF/5 to profile in the genomic DNA of the cell dilutions (Additional file 2: Table S2). We found a strong linear correlation between the theoretical and estimated dilution ratios up to a dilution of 1:100,000 (*R*^2^ = 0.9998) (Fig. 1b). Background noise observed at 0% PLC/PRF/5 cell input was < 0.0007% among 20 independent replicates, and the assay confidently detected a 0.001% fraction (Fig. 1c, d). To evaluate the biological noise of mutations from nontumor cells in the PLF sample, we profiled 20 mutations that were not detected in the matched tumor sample. We found background noise in all PLF clinical samples to be < 0.01%, which was used as the cutoff for MRD detection in the subsequent analysis (Fig. 1e).

**Fig. 2:**
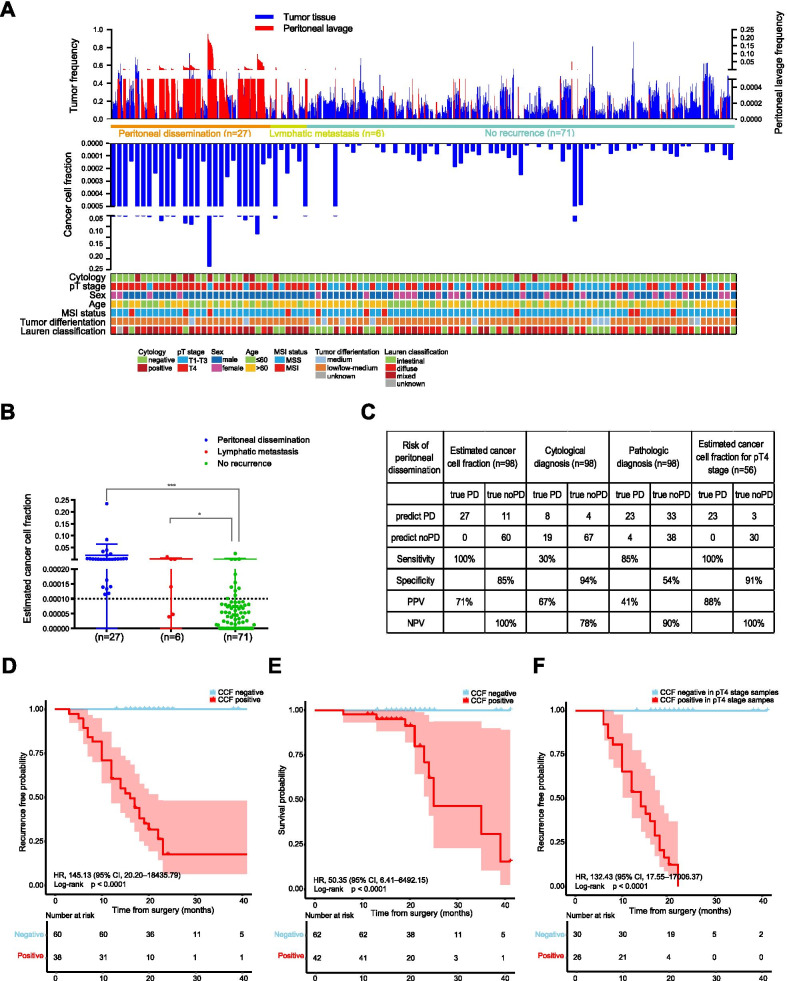
The performance of the cancer cell fraction model in gastric cancer patients. **a** Clinical and histopathologic parameters, somatic mutations and cancer cell fraction for all patients. Top panel, summary of the frequencies of the tracked mutations in tumor and matched PLF samples from 104 patients. Blue bar, the tumor frequency of each tracked mutation. Frequency values are shown on the left vertical axis. Red bar, the detected peritoneal lavage fluid frequency of each tracked mutation. Frequency values are shown on the right vertical axis. The clinical outcome of patients is indicated under the bar. Middle panel, the cancer cell fraction of each patient. Bottom panel, clinical and histopathological characteristics. **b** The distribution of the cancer cell fraction in patients with peritoneal dissemination (*n* = 27), lymphatic metastasis (*n* = 6) or no recurrence (*n* = 71). Reported *p* values were computed using a 2-tailed Wilcoxon Mann–Whitney U test. ***p < 0.001; *p < 0.05. **c** Binary results of the PLF mutation profiling model and clinical risk factors. Pathologic diagnosis was defined as high (pT4) or low (pT0–3) according to the standard criteria. PD, peritoneal dissemination; PPV, positive predictive value; NPV, negative predictive value. **d** The Kaplan–Meier survival analysis shows the probability of recurrence-free survival as determined by MRD analysis of PLF (*n* = 98). **e** Kaplan–Meier estimates of overall survival for 104 gastric cancer patients based on MRD analysis of PLF. **f** The Kaplan–Meier survival analysis shows the probability of recurrence-free survival (RFS) as determined by MRD analysis of PLF in stage pT4 patients (*n* = 56) (for peritoneal dissemination). Shaded areas in the Kaplan–Meier plots indicate 95% CIs. HR: hazard ratio; CCF: cancer cell fraction

We applied MRD analysis to a cohort of 104 GC patients with radical resection (Additional file 3: Figure S1; Additional file 2: Table S3). Based on exome sequencing of tumor tissue, 17 (median, range 3–23) mutations were selected and profiled on matched PLF samples (Additional file 2: Table S4, S5). The cancer cell fraction ranged from 0 to 23.41%, and 42 cases (40%) were MRD-positive. At the 41-month follow-up, 27 out of the 104 patients experienced PD, and all (100%) of them were MRD-positive. Six patients developed lymphatic metastasis, and 4 (67%) were MRD-positive (Fig. 2a, b; Additional file 2: Table S6). In addition, 15% (11/71) of the nonrecurrence patients were MRD-positive. The MRD analysis achieved 100% sensitivity, 85% specificity and 71% positive predictive value (PPV), and the MRD-positive patients showed a high risk of PD (HR = 145.13; 95% CI 20.20–18,435.79; *p* < 0.001) (Fig. 2c, d). In addition, MRD-positive patients were associated with decreased recurrence-free survival (RFS) and overall survival, and all 10 patients who died during the follow-up were MRD-positive patients with disease recurrence (Fig. 2d, e; Additional file 3: Figure S2A).

We further compared the predictive value of the MRD analysis with clinical risk factors (Additional file 3: Figure S2B, C). The MRD analysis showed the best performance (Fig. 2c) and was the strongest independent predictor of RFS for PD based on the Cox proportional hazard model (Additional file 2: Table S7). Interestingly, the MRD analysis exhibited a lower false-positive rate among stage pT4 patients (*n* = 56), delivering 100% (23/23) sensitivity, 91% (30/33) specificity and 88% (23/26) PPV (Fig. 2c, f).

In conclusion, our mutation-based MRD analysis exhibited highly improved performance compared with cytology or other PLF-based assays, making early detection and early intervention far before PDs become clinically detectable possible. This approach may help improve postoperative treatment to prevent PD and improve the overall survival of GC.

## Supplementary Information

**Additional file 1. **Materials and Methods.

**Additional file 2. Table S1**.Theoretical and estimated dilution ratio of cancer cells. **Table S2**. Detected mutations in standard curve. **Table S3**. Clinicopathological characteristics. **Table S4**. Summary of whole-exome sequencing and traced mutations. **Table S5**. Personalized somatic mutations in tumor tissue and paired PLF. **Table S6**. Raw data on the cancer cell fraction and clinical risk factors for the prediction of PD. **Table S7**. Recurrence-free survival analysis by clinicopathological variables and MRD analysis.

**Additional file 3. Fig. S1**.Patient enrollment, sample collection workflow and the prognosis prediction of patients. **Fig. S2**. Kaplan–Meier estimates of recurrence-free survival (RFS) based on clinical risk factors and MRD analysis. (A) Kaplan–Meier estimates of RFS based on MRD analysis in 104 gastric cancer patients. Peritoneal dissemination and lymphatic metastasis patients were combined as a recurrence group (*n* = 33). The Kaplan–Meier survival analysis shows the probability of RFS by (B) cytological diagnosis with peritoneal lavage fluid (*n* = 98) and (C) pT stage according to the 8th edition of the Union for International Cancer Control (*n* = 98) for peritoneal dissemination. A patient was classified as test-positive if the cytological diagnosis of peritoneal lavage fluid was positive (B) and the pathologic diagnosis was pT4 stage gastric cancer (C). CCF: cancer cell fraction. Shaded areas in the Kaplan–Meier plots indicate 95% CIs. HR: hazard ratio.

## Data Availability

The dataset supporting the conclusions of this article is available in the Genome Sequence Archive for Human repository [HRA000528 in https://bigd.big.ac.cn/gsa-human/].

## Abbreviations

PD: Peritoneal dissemination
GC: Gastric cancer
PLF: Peritoneal lavage fluid
HIPEC: Hyperthermic intraperitoneal chemotherapy
MRD: Minimal residual disease
cfDNA: Cell-free DNA
PPV: Positive predictive value
RFS: Recurrence-free survival
