# Supplementary material for: Personalized analysis of minimal residual cancer cells in peritoneal lavage fluid predicts peritoneal dissemination of gastric cancer
Source: J Hematol Oncol. 2021 Oct 12;14:164. doi: 10.1186/s13045-021-01175-2 (PMC8507390; doi:10.1186/s13045-021-01175-2)
Supplement: Supplementary file 1 — Additional file 1. Materials and Methods. [file 13045_2021_1175_MOESM1_ESM.docx]

**Materials and Methods**

**Patients and samples**

From June 2017 to December 2019, 110 gastric cancer patients (mean [SD] age, 61 [9.6] years; 79 [76%] male) scheduled for surgery in the National Cancer Center, Cancer Hospital, Chinese Academy of Medical Sciences were enrolled in our study. Eligibility criteria of the study included a diagnosis of stage I to III resectable gastric cancer determined by chest-abdominal-pelvic enhanced computed tomography-scan (CT-scan), MRI or upper gastrointestinal endoscopy ± endoscopic ultrasound (EUS); no metastatic disease evident on staging computed tomography (CT); age < 80 years; no preoperative radiotherapy or chemotherapy; and a treatment plan for resection. In accordance with NCCN guidelines, postoperative chemotherapy was recommended after surgery. Patients with another malignant neoplasm diagnosed within the last 3 years were excluded. After therapy, surveillance was performed according to the standard of care, with clinical assessment every 3 months for the first 2 years and every 6 months for the subsequent years. The primary endpoint of the study is clinical detection of disease recurrence or metastasis, including local recurrence, PD, hematogenous metastasis and lymphatic metastasis. TNM stages were determined according to the 8th edition of the Union for International Cancer Control. Finally, 6 patients were excluded from the study for palliative surgery (*n* = 4) or the presence of other types of cancer (*n* = 2). A total of 104 patients were included in the analysis (Additional file 3: Fig. S1).

Recurrence of PD was defined as lesions detected in one of the following ways: MRI, CT scan, PET-CT scan visualized [^18^F]fluorodeoxyglucose (^18^F-FDG) uptake over peritoneum and bowel with or without ascites > 50 mL, or tumor cells detected in ascites or biopsy of peritoneal lesions. The median follow-up period was 20 months (6–41 months). The clinicopathological characteristics of the 104 patients are listed in Additional file 2: Table S3. Surgery consisted of radical resection of the primary tumor and at least D2 lymph node dissection.

For collection of PLF samples, 300–400 mL of normal saline was used to wash the upper abdominal cavity after abdominal exploration and before any manipulation of the tumor during surgery. A total of 200 mL of PLF was collected. PLF (100 mL) was examined through conventional cytological diagnosis with Papanicolaou and Giemsa staining according to the NCCN and EMSO guidelines to evaluate peritoneal free cancer cells [1, 2]. The remaining 100 mL of PLF was used in mutation profiling to compare with the standard of care solution. PLF samples were centrifuged first at 2500 rpm (1098 g) for 10 min to remove the supernatant and then at 12,000 rpm for 10 min to obtain the PLF pellet. Blood samples were collected before surgery through a standard blood draw protocol. Fresh tumor tissue was collected from surgery. Tumor tissues, matched white blood cells (WBCs) and PLF pellets were stored at -80 °C until DNA extraction. In total, PLF, frozen tumor tissue and blood samples were collected for analysis from 104 patients.

**Construction and validation of the cancer cell fraction model**

Due to the low fraction of residual cancer cells among normal cells in PLF, the number of molecules present for a given mutation site in the sequencing (distinct coverage) might be insufficient to precisely detect the mutation. Thus, the detected frequency of one mutation might not precisely represent the ratio of cancer cells [3, 4]. Here, we set up a model to estimate the cancer cell ratio based on allele frequency and sequencing depth of somatic mutations in tumor tissue and paired PLF samples (Fig. 1A). We performed exome sequencing on the DNA from tumor tissue and matched WBC samples to identify somatic mutations and designed customized primers to profile 20 somatic mutations in the peritoneal lavage samples with Mutation Capsule technology [5]. For each traced somatic mutation, we determined the mutation frequency in the PLF sample and compared it with the frequency in the primary tumor. The sample-level estimated cancer cell fraction was determined with the maximum likelihood estimation.

**Cell lines and standard reference**

To validate the accuracy of the cancer cell fraction model, two cell lines (PLC/PRF/5 and A549) used to construct the cancer cell fraction model were obtained from the Cell Bank of the Chinese Academy of Sciences (Shanghai, China). In the model, PLC cells were used as the “cancer cells” and diluted with A549 cells, which were used as the “control cells”. The two cell lines were counted with FACSAriaSORP flow cytometry (Becton Dickinson; San Diego, CA, USA) and serially diluted for a total of 9 dilutions (PLC cell fraction = 0%, 0.0001%, 0.0003%, 0.001%, 0.005%, 0.05%, 0.5%, 5% and 33%). The details of the model construction and number of cells are shown in Additional file 2: Table S1.

**DNA extraction and library preparation**

Genomic DNA was extracted from PLF, frozen tumor tissue, WBC and cell line samples using the QIAamp DNA Mini Kit (Qiagen; Hilden, Germany). Exome sequencing was performed on genomic DNA (1000 ng) from frozen tumor tissue and WBCs, and a customized panel targeting 20 mutations was performed on genomic DNA (200 ng) from cell lines or PLF. Genomic DNA was sheared into small fragments (mean length ~ 200 bp) with the Covaris E220 instrument (Covaris; Woburn, MA, USA). The sheared DNA was prepared for genomic libraries using the KAPA Hyper Prep Kit (Roche; Basel, Switzerland) through a series of enzymatic steps, including end repair, dA tailing, ligation of adaptors and amplification, following standard protocols except for the use of a customized adaptor with barcodes for PLF and cell line samples as previously described [5, 6].

**Whole-exome sequencing and identification of somatic mutations**

Whole-genome libraries of tumor and matched WBC DNAs were enriched for exome regions with Agilent SureSelectXT Human All Exon V5 probe and reagents (Agilent; Santa Clara, CA, USA). The captured and amplified libraries were sequenced on the Illumina HiSeqX Ten with 150-bp paired-end sequencing to a median depth of 183× for tumor tissue samples and 105× for WBC samples after removing duplicate molecules. The sequencing raw data (FASTQ file) were aligned to the UCSC human reference genome hg19 using Burrows-Wheeler aligner software (BWA, v0.7.15). Basic processing, marking duplicates, local realignments and score recalibration were analyzed using the Genome Analysis Toolkit (GATK, v3.6), Picard (v2.7.1) and Samtools (v1.3.1). Candidate somatic mutations were detected by comparing sequencing data from tumor tissue samples with MuTect1 and Strelka. All selected mutations were further validated with manual inspection using Integrated Genome Viewer (IGV) [7]. A median of 101 mutations were detected in the primary tumor samples of each patient.

**Customized assay to profile multiple mutations**

Genomic DNA (200 ng) from cell lines or PLF was used for the customized panel targeting 20 mutations. The ligation products with customized adaptors were amplified for a whole-genome library and used as templates to profile mutations identified in the matched tumor tissue. The customized adaptor contained sufficient unique DNA barcodes identifying each original molecule [5]. We selected 20 tumor-specific somatic mutations from the whole-exome sequencing results of tumor tissue for primer design. For each patient, the 20 tumor-specific variants were selected according to the potential to be a driver mutation, the confidence of the mutation by IGV and the frequency of the mutation. Oligo software (v7.53) was used to design multiplex PCR primer pairs for the two rounds of nested amplification and verified uniqueness in the human genome (http://genome.ucsc.edu/) to ensure amplification efficiency. In the first round of amplification, the target regions were amplified in 9 cycles of PCR using a target-specific primer and a primer matching the adapter sequence. A second round of 14 cycles of amplification was performed with one pair of nested primers matching the adapter and the target region to further enrich the target region and add the Illumina adapter sequences to the construct. This method can amplify more than 200 target regions in parallel. The targeted sequencing libraries were sequenced on an Illumina NovaSeq 6000 with a median depth of 101,703× before deduplication and 11,760× after removing duplicate molecules. The median on-target ratio of reads mapped to the target region was 80%. For the 104 patients, we chose a total of 1,717 mutations in the analysis. On average, 17 somatic mutations (3–23 mutations per person) were tracked for each patient (Additional file 2: Table S4, 5).

**Bioinformatics pipeline and SNV calling**

With the DNA barcode in the customized adapter, redundant reads can be tracked from an original DNA molecule to minimize false-positive calling due to PCR amplification and sequencing errors. Sequencing reads were mapped to the hg19 reference genome using 'bwa mem' with the default parameters after extracting tags and removing sequence adapters. The SNV analysis mutation frequency calculation method was used as previously described [5]. Briefly, reads with the same tags and start and end coordinates were grouped into unique identifier families (UID families). If > 80% of the reads in a UID family harbor the same mutation identified in the matched tumor tissue, the UID family was defined as an effective unique identifier family (EUID family) with the mutation. The mutant EUID families were further confirmed by manual inspection (IGV) and used to calculate the frequency of the mutation with the total number of UID families covering the mutant site. Finally, 25.7% (441/1,717) of the tracked mutations were detected in matched PLF samples.

**PLF mutation profiling model**

An estimated cancer cell fraction analysis was performed in the R statistical environment version 3.6.3.

Assumption: 1. Because of the low fraction of residual cancer cells among normal cells in PLF, there are chances that some mutations present in the tumor tissue are not detected in the corresponding PLF sample. The algorithm is also based on the assumption that this tumor-PLF mismatch results from the low concentration of mutant molecules and/or the randomness in sampling. 2. The estimation model is based on the assumption that mutant allele reads and nonmutant wild-type allele reads fit well with the binomial distribution.

Let ($m_{1},m_{2},\ldots,m_{n-1},m_{n}$) be N mutations in PLF, each with a mutation frequency of ${Pc}_{i},$ sequencing depth of $D_{i}$ and mutation read number of $A_{i}$. ${Pt}_{i}$ indicates the mutation frequency in the corresponding solid tumor tissue. If R is the overall cancer cell fraction of this sample, then for mutation $m_{i}$, the mutational frequency in PLF,${Pc}_{i}$ = ${Pt}_{i}*R,$ and the distribution of observed $X_{i}$ reads with mutation $m_{i}$ out of $D_{i}$ reads in PLF follow the binomial distribution:

$$X_{i}\sim B(D_{i},{Pc}_{i})$$

Then, the probability of observing $A_{i}$ reads with mutation $m_{i}$ out of $D_{i}$ reads at position $m_{i}$ is:

$$P_{i}(X_{i}=A_{i})=\binom{D_{i}}{A_{i}}{{Pc}_{i}}^{A_{i}}{(1-{Pc}_{i})}^{D_{i}-A_{i}}$$

Assuming that the events of observing ($A_{1}, A_{2},\ldots,A_{n-1},A_{n}$) reads support that mutations ($m_{1},m_{2},\ldots,m_{n-1},m_{n}$) are independent, then the probability of observing a sequence of ($A_{1}, A_{2},\ldots,A_{n-1},A_{n}$) is the product of $P_{i}(X_{i}=A_{i})$ for each mutation $m_{i}$. Let $P$ be the likelihood function of cancer cell fraction $R$:

$$L\left（ R \right）=P=\prod_{i=1}^{n} P_{i}=\prod_{i=1}^{n} \binom{D_{i}}{A_{i}}{{(Pt}_{i}*R)}^{A_{i}}{(1-{Pt}_{i}*R)}^{D_{i}-A_{i}}$$

$P$ is maximized to obtain the predicted $R$, namely, $\hat{R}$:

$$\hat{R}=\arg{max \atop R}\prod_{i=1}^{n} P_{i}=\prod_{i=1}^{n} \binom{D_{i}}{A_{i}}{{(Pt}_{i}*R)}^{A_{i}}{(1-{Pt}_{i}*R)}^{D_{i}-A_{i}}$$

$$=\arg{max \atop R}ln\left[ \prod_{i=1}^{n} \binom{D_{i}}{A_{i}}{{(Pt}_{i}*R)}^{A_{i}}{(1-{Pt}_{i}*R)}^{D_{i}-A_{i}} \right]$$

$$=\arg{max \atop R}\sum_{i=1}^{n} \left[ ln\binom{D_{i}}{A_{i}}+A_{i}*ln{(Pt}_{i}*R)+(D_{i}-A_{i})*ln(1-{Pt}_{i}*R) \right]$$

A search through a grid (0.00001, 1, step = 0.00001) for $R$ in the above formula yields the optimized $\hat{R}$. Maximum mutation frequencies in tumor tissues were used for normalization of tumor sample purity.

**Statistical analysis**

Recurrence-free survival (RFS) was calculated as the time from surgery to the date of recurrence, last visit or death, whichever came first. Overall survival (OS) was measured from surgery to the date of cause-specific death or last visit. Survival curves were plotted and analyzed with the Kaplan–Meier method, and the log-rank test was used to test the significance of the difference between survival curves.

In addition to the cancer cell fraction, the prognostic value of other factors, such as Lauren classification, pathology T/N stage, cytology, and lymphovascular and nerve invasion, was also analyzed. HRs were first estimated using univariate Cox proportional hazard models. Thereafter, factors with statistical significance (*p* < 0.05) were further assessed in a multivariate analysis using the Cox proportional hazard model. Whenever there was no event in one group of categorical variables, Firth's penalized likelihood was adopted to allow for monotone likelihood (R package "coxphf"). All statistical analyses were performed using the survival package or coxphf package from R software (V.3.6.3). Statistical comparison of the cancer cell fraction distribution in patients with no recurrence, peritoneal dissemination or lymphatic metastasis was performed using a 2-tailed Wilcoxon Mann–Whitney U test with a significance level set at 5%, and the results are expressed as the mean ± SD. *P* < 0.05 was considered statistically significant.

**REFERENCES**

1. National Comprehensive Cancer Network. Gastric Cancer. Version 2.2021. Accessed March 9, 2021.

2. Cunningham, . D, Verheij, M., Smyth, E., et al. Gastric cancer: ESMO Clinical Practice Guidelines for diagnosis, treatment and follow-up. 2016.

3. Patel JP, Gönen M, Figueroa ME, Fernandez H, Sun Z, Racevskis J, et al. Prognostic relevance of integrated genetic profiling in acute myeloid leukemia. N Engl J Med. 2012;366(12):1079-89.

4. Schuurhuis GJ, Heuser M, Freeman S, Béné MC, Buccisano F, Cloos J, et al. Minimal/measurable residual disease in AML: a consensus document from the European LeukemiaNet MRD Working Party. Blood. 2018;131(12):1275-91.

5. Qu C, Wang Y, Wang P, Chen K, Wang M, Zeng H, et al. Detection of early-stage hepatocellular carcinoma in asymptomatic HBsAg-seropositive individuals by liquid biopsy. Proc Natl Acad Sci U S A. 2019;116(13):6308-12.

6. Zhang W, He H, Zang M, Wu Q, Zhao H, Lu L-l, et al. Genetic Features of Aflatoxin-Associated Hepatocellular Carcinoma. Gastroenterology. 2017;153(1):249-62.e2.

7. Fujikura K, Hosoda W, Felsenstein M, Song Q, Reiter JG, Zheng L, et al. Multiregion whole-exome sequencing of intraductal papillary mucinous neoplasms reveals frequent somatic KLF4 mutations predominantly in low-grade regions. Gut. 2020.
