# Supplementary material for: Personalized analysis of minimal residual cancer cells in peritoneal lavage fluid predicts peritoneal dissemination of gastric cancer
Source: J Hematol Oncol. 2021 Oct 12;14:164. doi: 10.1186/s13045-021-01175-2 (PMC8507390; doi:10.1186/s13045-021-01175-2)

Supplementary Figures

Fig. S1. Patient enrollment, sample collection workflow and the prognosis prediction of patients

Fig. S2. Kaplan–Meier estimates of recurrence-free survival (RFS) based on clinical risk factors and MRD analysis

Fig. S1. Patient enrollment, sample collection workflow and the prognosis prediction of patients.

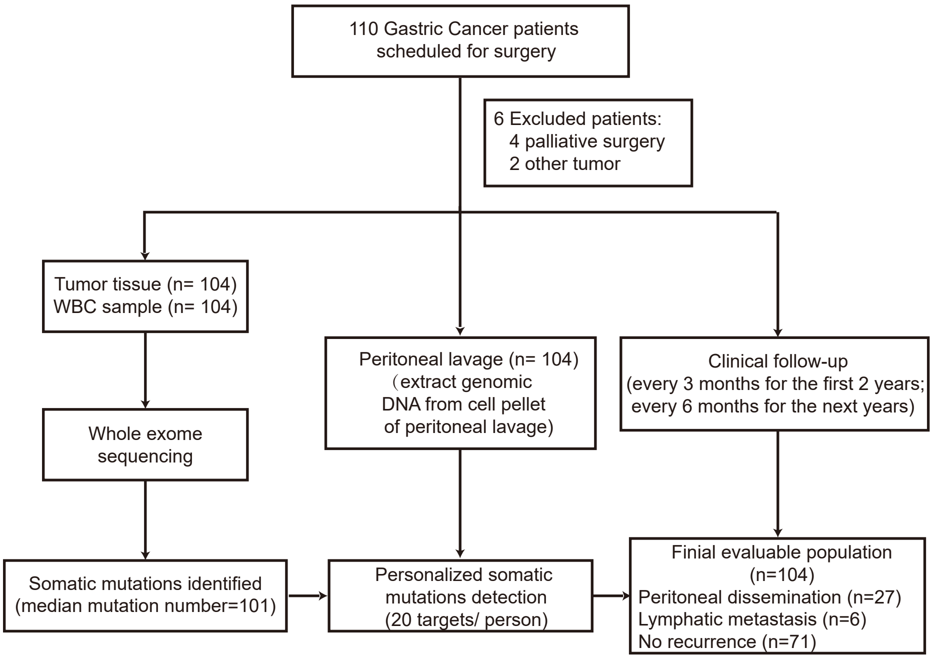

Figure S2. Kaplan–Meier estimates of recurrence-free survival (RFS) based on clinical risk factors and MRD analysis.

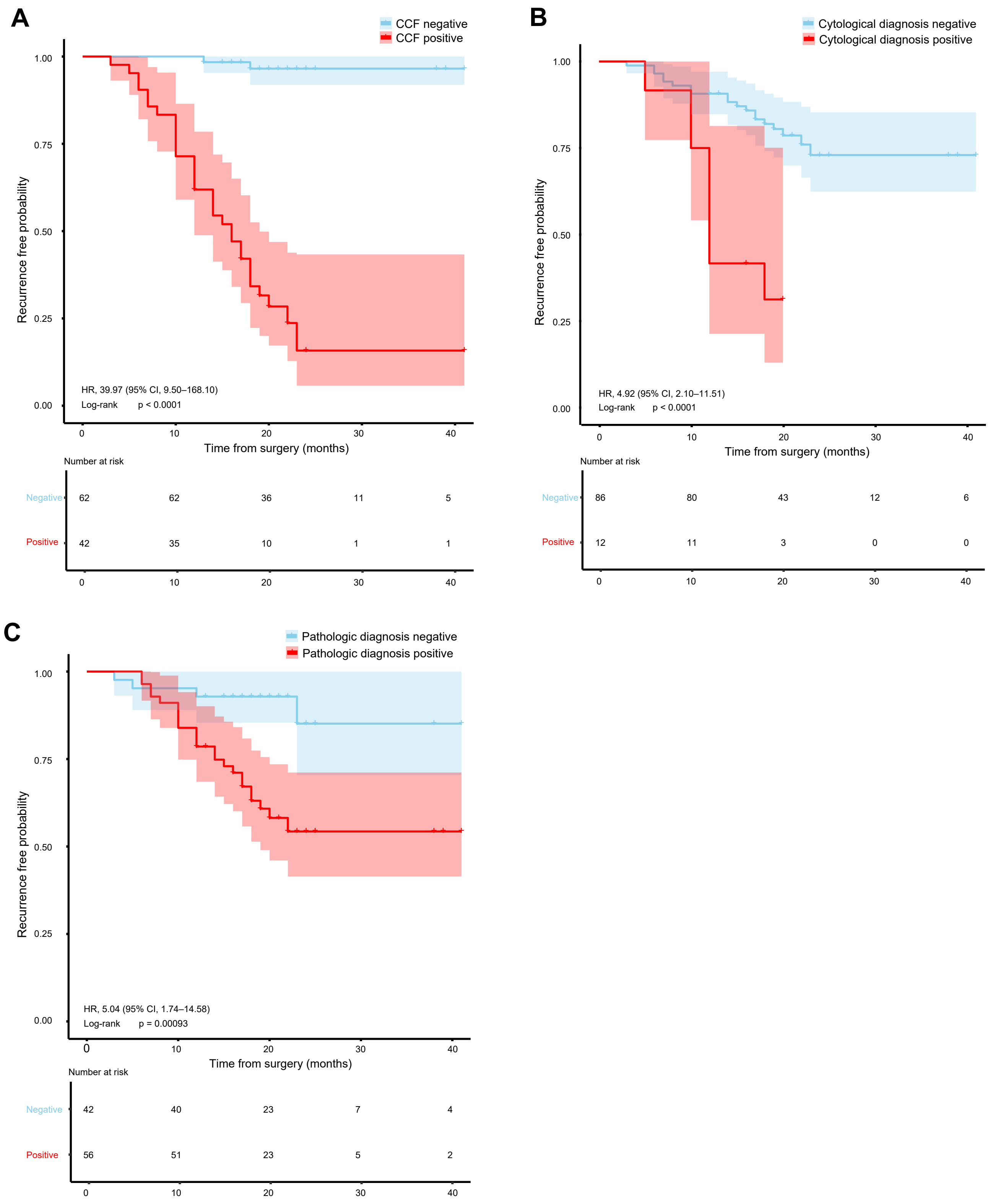

Supplement: Supplementary file 3 — Additional file 3. Fig. S1.Patient enrollment, sample collection workflow and the prognosis prediction of patients. Fig. S2. Kaplan–Meier estimates of recurrence-free survival (RFS) based on clinical risk factors and MRD analysis. (A) Kaplan–Meier estimates of RFS based on MRD analysis in 104 gastric cancer patients. Peritoneal dissemination and lymphatic metastasis patients were combined as a recurrence group (n = 33). The Kaplan–Meier survival analysis shows the probability of RFS by (B) cytological diagnosis with peritoneal lavage fluid (n = 98) and (C) pT stage according to the 8th edition of the Union for International Cancer Control (n = 98) for peritoneal dissemination. A patient was classified as test-positive if the cytological diagnosis of peritoneal lavage fluid was positive (B) and the pathologic diagnosis was pT4 stage gastric cancer (C). CCF: cancer cell fraction. Shaded areas in the Kaplan–Meier plots indicate 95% CIs. HR: hazard ratio. [file 13045_2021_1175_MOESM3_ESM.pdf]
